# RETRACTION: The Impact of Psychological Interventions on Surgical Site Wound Healing Post‐Surgery in Psoriasis Patients: A Meta‐Analysis

**DOI:** 10.1111/iwj.70568

**Published:** 2025-04-18

## Abstract

**RETRACTION:** WangN. , PeiL. , ZhangM. , WangG. , ZhengS. , KouX. , ChenH. , “The Impact of Psychological Interventions on Surgical Site Wound Healing Post‐Surgery in Psoriasis Patients: A Meta‐Analysis,” International Wound Journal 21, no. 4 (2024): e14509, 10.1111/iwj.14509.38151959 PMC10958094

The above article, published online on 27 December 2023, in Wiley Online Library (http://onlinelibrary.wiley.com/), has been retracted by agreement between the journal Editor in Chief, Professor Keith Harding; and John Wiley & Sons Ltd. Following an investigation by the publisher, all parties have concluded that this article was accepted solely on the basis of a compromised peer review process. The editors have therefore decided to retract the article. The authors did not respond to our notice regarding the retraction.

**RETRACTION:** N. Wang , L. Pei , M. Zhang , G. Wang , S. Zheng , X. Kou , H. Chen , “The Impact of Psychological Interventions on Surgical Site Wound Healing Post‐Surgery in Psoriasis Patients: A Meta‐Analysis,” International Wound Journal 21, no. 4 (2024): e14509, 10.1111/iwj.14509.38151959 PMC10958094

The above article, published online on 27 December 2023, in Wiley Online Library (http://onlinelibrary.wiley.com/), has been retracted by agreement between the journal Editor in Chief, Professor Keith Harding; and John Wiley & Sons Ltd. Following an investigation by the publisher, all parties have concluded that this article was accepted solely on the basis of a compromised peer review process. The editors have therefore decided to retract the article. The authors did not respond to our notice regarding the retraction.

